# Magnetic Properties of Iron Oxide Nanoparticles Do Not Essentially Contribute to Ferrogel Biocompatibility

**DOI:** 10.3390/nano11041041

**Published:** 2021-04-19

**Authors:** Felix A. Blyakhman, Alexander P. Safronov, Emilia B. Makarova, Fedor A. Fadeyev, Tatyana F. Shklyar, Pavel A. Shabadrov, Sergio Fernandez Armas, Galina V. Kurlyandskaya

**Affiliations:** 1 Institute of Natural Sciences and Mathematics, Ural State Medical University, 620028 Ekaterinburg, Russia; feliks.blyakhman@urfu.ru (F.A.B.); emilia1907@yandex.ru (E.B.M.); fdf79@mail.ru (F.A.F.); t.f.shkliar@urfu.ru (T.F.S.); pavel.shabadrov@urfu.ru (P.A.S.); 2 Institute of Natural Sciences and Mathematics, Ural Federal University, 620002 Ekaterinburg, Russia; alexander.safronov@urfu.ru; 3 Institute of Electrophysics UB RAS, 620016 Ekaterinburg, Russia; 4 Ural Scientific Institute of Traumatology and Orthopaedics, 620014 Ekaterinburg, Russia; 5 Institute for Medical Cell Technologies, 620026 Ekaterinburg, Russia; 6 SGIKER, Universidad del País Vasco UPV/EHU, 48080 Bilbao, Spain; sergio.fernandez@ehu.eus; 7 Departamento de Electricidad y Electrónica, Universidad del País Vasco UPV/EHU, 48940 Leioa, Spain

**Keywords:** hydrogel, Fe_2_O_3_ and Al_2_O_3_ nanoparticles, gel-based composites, magnetic properties, cells, biocompatibility

## Abstract

Two series of composite polyacrylamide (PAAm) gels with embedded superparamagnetic Fe_2_O_3_ or diamagnetic Al_2_O_3_ nanoparticles were synthesized, aiming to study the direct contribution of the magnetic interactions to the ferrogel biocompatibility. The proliferative activity was estimated for the case of human dermal fibroblast culture grown onto the surfaces of these types of substrates. Spherical non-agglomerated nanoparticles (NPs) of 20–40 nm in diameter were prepared by laser target evaporation (LTE) electrophysical technique. The concentration of the NPs in gel was fixed at 0.0, 0.3, 0.6, or 1.2 wt.%. Mechanical, electrical, and magnetic properties of composite gels were characterized by the dependence of Young’s modulus, electrical potential, magnetization measurements on the content of embedded NPs. The fibroblast monolayer density grown onto the surface of composite substrates was considered as an indicator of the material biocompatibility after 96 h of incubation. Regardless of the superparamagnetic or diamagnetic nature of nanoparticles, the increase in their concentration in the PAAm composite provided a parallel increase in the cell culture proliferation when grown onto the surface of composite substrates. The effects of cell interaction with the nanostructured surface of composites are discussed in order to explain the results.

## 1. Introduction

In the past several decades, a number of biologically inert materials were introduced as useful composites for cell culturing for the needs of tissue engineering [1,2]. Among others, magnetic soft materials such as ferrogels (FG) have demonstrated promising applications in biomedicine due to the ability to change their physical properties in response to an external magnetic field [3,4] or to be used as the components of the addressed delivery by the gradient external magnetic field both the encapsulated drugs and soft implants [5]. Furthermore, a magnetic field *per se* may stimulate the biological activity of certain types of cells [6,7] by the enhancement of cell adhesion, proliferation, differentiation as far as modifying properties of the fluids used for cell cultivation [8,9,10].

In our earlier studies, we tested the biocompatibility of ferrogel based on the polyacrylamide (PAAm) polymer network with embedded maghemite (γ-Fe_2_O_3_) magnetic nanoparticles (MNPs) fabricated by the electrophysical technique of the laser target evaporation (LTE) [11]. Such parameters as adhesion and proliferation of human peripheral leucocytes or human dermal fibroblasts when grown onto the surface of the FG samples were studied. It became clear that the addition of magnetic nanoparticles to the PAAm gel network always resulted in an increase in cell adhesion and proliferation when grown onto the surface of the gel-based composites [12,13]. In particular, the gradual increase in magnetic nanoparticle concentration in PAAm gel from 0 to 2 wt.% was accompanied by the increase in cell monolayer density by a factor of five for the culture grown onto the FG surface [14]. 

Meanwhile, the nature of the positive impact of MNPs on the biocompatibility of ferrogels is still not clear. Many direct and/or indirect factors can contribute to this phenomenon. In particular, the addition of magnetic nanoparticles to the network of PAAm gel significantly changes the electrical and mechanical properties of FG that strongly control the cell adhesion and proliferation on the ferrogel substrate [13,14,15]. The arrangement of MNPs in ferrogel structure is also a potential source of stray magnetic fields [5,16]. This feature, could hypothetically influence biological cell activity as well. At a time, the existence of stray magnetic fields of the gel-based composites can be used for the definition of implant position or degradation state using magnetic field sensors [5,16].

The aim of the present study was to check whether the magnetic MNPs per se affect the ferrogel biocompatibility. In particular, we intended to compare the biocompatibility of series of composite gels with embedded superparamagnetic and diamagnetic nanoparticles, which were almost the same in terms of their characteristic dimensions, morphology, and the properties of their suspensions in water. We analyzed the mechanical properties of these series of composite gels, their electrical potential, and the proliferative activity of human dermal fibroblasts on the surface of these two series of gel-based substrates.

Here, we show that regardless of the superparamagnetic or diamagnetic nature of nanoparticles, the increase in NPs concentration in the nanostructured PAAm gel was accompanied by a similar increase in the cell proliferation on the surface of the gel-based substrates.

## 2. Materials and Methods

### 2.1. Synthesis and Characterization of Nanoparticles

Maghemite superparamagnetic (γ-Fe_2_O_3_) and alumina (Al_2_O_3_) diamagnetic nanoparticles, denoted as MNPs (maghemite) and ANPs (alumina), were accordingly synthesized by means of laser target evaporation (LTE) method. The main details of the fabrication technology and the apparatus of LTE were described in detail in our previous reports [5,11,13]. As a source of laser irradiation for iron oxide rotating target evaporation, we used a Ytterbium (Yb) fiber laser at a 1.07 µm wavelength.

As-synthesized air-dry MNPs and ANPs were studied using transmission electron microscopy (JEOL JEM2100, JEOL Corporation, Tokyo, Japan) operated at 200 kV. For TEM studies MNPs and ANPs were spread onto a Cu grid. Characterization of phase composition of MNPs and ANPs was done using X-ray diffraction technique (Bruker D8 Discover, Bruker Corporation, Billerica, MA, USA) with a graphite monochromator (Cu K_α_ radiation, wavelength λ = 1.5418 A) and a scintillation detector. The diffractograms were processed by Rietveld full-profile refinement using built-in Bruker software TOPAS-3. The specific surface area (S_sp_) of nanoparticles was measured using Micromeritics TriStar3000 analyzer (Micromeritics, Norcross, GA, USA).

Magnetic characterization of MNPs and ANPs was done using a superconducting quantum device, SQUID (Quantum Design MPMS-7, Quantum Design Inc., San Diego, CA, USA). All magnetic measurements were made at room temperature. Apart from the measurements of the magnetic hysteresis loops M(H) for MNPs and ANPs, M(H) loops were also measured for all kinds of gels (blank gel, ferrogel field with MNPs, and gels filled with ANPs). For magnetic measurements of MNPs and ANPs, samples of about 5 mg for MNPs and 10 mg for AMPs were used. For gel-based composites, samples of about 60 mg were employed. 

The mean hydrodynamic diameter of particles/aggregates in suspensions was measured by the dynamic light scattering (DLS) using Brookhaven ZetaPlus analyzer (Brookhaven Instruments, Holtsville, NY, USA). The same instrument was used for the measurement of the zeta-potential in suspensions by the electrophoretic light scattering (ELS).

In addition, scanning electron microscopy (SEM) of dried MNPs and ANPs filled gel composites were performed with 20 kV accelerating voltage (JEOL JSM-640, JEOL Corporation, Tokyo, Japan). In order to avoid the surface charging of the polymer composite, a carbon film using a sputtering technique was deposited onto the composite surfaces with a thickness of about 20 nm.

### 2.2. Synthesis of Composite Gels

Synthesis of composite gels with embedded MNPs and ANPs was performed by free-radical polymerization of acrylamide monomers (AAm, AppliChem, Darmstadt, Germany) dissolved in water suspensions of MNPs and ANPs. The suspensions were prepared by the dispersion of nanoparticles in 5 mM water solution of sodium citrate, which was an electrostatic stabilizer of the suspension. Dispersions were de-aggregated by ultrasound treatment with permanent cooling for 30 min using Cole-Parmer CPX-750 (Cole-Parmer, Vernon Hills, IL, USA) processor operated at 250 W. Remained large aggregates were precipitated by centrifuging for 5 min at 8000 rpm using Hermle Z383 centrifuge (Hermle AG, Gosheim, Germany). De-aggregation in suspensions was controlled using DLS. The concentration of nanoparticles in these stock suspensions was determined using the weight of a dry residue after drying at 90 °C to the constant weight (with the correction for the dissolved sodium citrate). The content of MNPs in the stock suspension was as high as 4.8% by weight, and the content of ANPs was as high as 4.0%.

Stock suspensions were diluted by 5 mM water solution of sodium citrate to provide variation of nanoparticle content in the resulted composite gels. Monomer AAm was dissolved in MNPs and ANPs suspensions in 1.6 M concentration. Cross-linking agent N,N’-methylene bisacrylamide (MBAA, Merck Schuchardt, Hohenbrunn, Germany) was added in 1:100 molar ratio to AAm. An initiator, ammonium persulfate (APS), was used at a 3 mM concentration. Polymerization was performed at room temperature employing N,N,N’,N’-tetra-ethylene-methylenediamine (TEMED, Merck Schuchardt, Hohenbrunn, Germany) in 5 mM concentration as a catalyst.

For the implication of substrates for cell cultivation, the composite gels were synthesized in the shape of thin sheets. Therefore, polymerization was done between two polished glass plates, separated by 0.8 mm spacers and using the mold sealing by a silicon resin. The reaction mixture was poured between the plates using a syringe. It took approximately 5 min for the gelation of the reaction mixture in the mold. The mold was kept for an extra 60 min to complete the polymerization, and then it was disassembled. The resulted sheets of composite gels with embedded MNPs and ANPs were extensively washed in distilled water with daily water renewal in order to remove salts and unreacted monomer until the equilibrium water uptake was achieved. The final contents of nanoparticles in the composite gels swollen to equilibrium were as high as 0.33%, 0.63%, and 1.19% in the gel series with embedded MNPs, and 0.34%, 0.61%, and 1.23% in the gel series with embedded ANPs.

Afterward, the gel sheets were equilibrated for 2 days in Hanks Balanced Salt Solution (HBSS) pH = 6.8–7.2 (PanEco Ltd. Moscow, RF, Russia) with gentamicin (100 mg/L) with a daily renewal of the solution. Then for 2 days, they were kept in 199 solution pH = 7.0–7.4, osmolality 300 ± 20 mmol/kg, and a buffering capacity of ≤ 1.5 mL (PanEco Ltd. Moscow, RF, Russia) with gentamicin (100 mg/L) with daily renewal. Then the substrates in the shape of disks (13 mm in diameter) were cut from the gel sheets to fit the wells of the standard 24-well polystyrene plate for cell culturing. Prior to their use in the cell culture, the gel-based substrates were sterilized in an autoclave at 121 °C for 20 min.

### 2.3. Mechanical Properties and Electrical Potential of Nanostructured Gels

The elastic properties of gels and FGs were determined using a laboratory setup for mechanical tests [12,15]. Cylindrical samples were placed between two plates. One was connected rigidly to the actuator of a linear electromagnetic motor, and the other was connected to a precision strain-gage sensor. The motor induced compression strain with a magnitude of up to 20% in steps of 2% of the initial gel length. Stress–strain dependences were plotted as a result of these tests, and their linear sections were used to determine the Young modulus for the investigated materials. 

The electrical potential of gels was determined using the standard technique, which is routinely applied to living cells. Specifically, two identical silver-chloride electrodes in glass micropipettes TW150F-6 (World Precision Instruments, Sarasota, FL, USA) with a tip diameter of ~1 μm filled with a 3 M KCl solution were used. One electrode was placed in the solution surrounding the gel, and the other one was introduced into the studied sample. The potential difference was measured with an INA 129 instrumentation amplifier (Burr-Brown, Dallas, TX, USA).

### 2.4. Human Dermal Fibroblasts Culture 

The lines of human dermal fibroblasts were obtained from donor skin, as described previously [10,13,14]. Briefly, tissue biopsies were collected from patients (who have given the informed contest) during surgery. The study was approved by the Ethics Committee of the Institute of Medical Cell Technologies, Ekaterinburg. Skin biopsies were cut into small pieces, and cells were extracted by tissue dissociation method. Extracted fibroblasts were grown in culture flasks (Nunc, Roskilde, Denmark) at 37 °C and 5% CO_2_. Cells were passaged when they covered 80% of the flask surface area. Then sub-culturing cells were treated with 0.25% trypsin-EDTA solution (Gibco, Thermo Fisher Scientific, Inc., Waltham, MA, USA). Cell number was estimated by cell counter TC-20 device (Bio-rad, Hercules, CA, USA). The cell viability was measured by staining with trypan blue. Fibroblasts were stored in liquid nitrogen.

### 2.5. Cells Proliferation Assays on Nanostructured Gel-Based Composites

Thawed cells were passaged 2 times before they were used in experiments. Fibroblasts (fifth passage) were detached from the flask surface by trypsin. After detachment of cells, trypsin was neutralized by fetal bovine serum. Gel discs with MNPs, ANPs, and blank gel discs were placed into the wells of 24-well tissue-treated culture plates (Nunc, Roskilde, Denmark). Some wells were left empty to be used as controls of cell growth on tissue-treated culture plastic. Fibroblasts were re-suspended in a growth medium. The suspension was dispensed in wells with a seeding density of 3000 viable cells/cm^2^ (viability ≥ 95%) for all types of substrates. Plates were incubated at 37 °C, in the 5% CO_2_ atmosphere for 96 h. All cell experiments were performed without the application of the external magnetic field. However, we did not shield possible laboratory magnetic fields, which usually do not exceed one Oersted strength. After incubation, cells in the monolayer were fixed with 2.5% glutaric aldehyde. Fibroblasts on the substrate surfaces were visualized by staining cell nuclei with 4’,6-diamidino-2-phenylindole (DAPI, Sigma-Aldrich, St. Louis, MO, USA) and cytoplasm with 0.3% pyrazolone yellow solution. Cells were counted using a fluorescent Axio Lab A1 FL (Carl Zeiss, Oberkochen, Germany) microscope. The analysis for nine fields of view for each sample at “×100” magnification was done. The number of cells in images was estimated using the ImageJ software (Wayne Rasband, NIH, Bethesda, MD, USA).

The experiment was performed in 6 replicates. We used the non-parametric Mann–Whitney U-test in order to compare the statistical significance of the difference between two independent groups with a level of significance set at 0.05. Statistical data processing was performed employing the application software package “STATISTICA 6.0” (Statsoft, Dell, Round Rock, TX, USA).

## 3. Results

### 3.1. Properties of Nanoparticles and Precursor Suspensions

**Figure 1:**
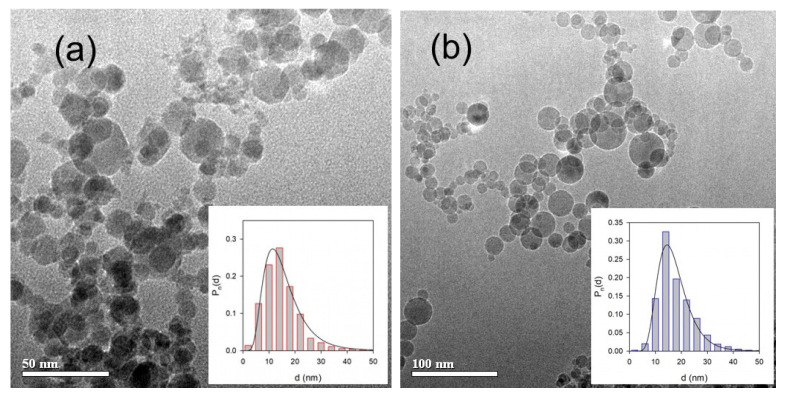
TEM images of LTE MNPs (**a**) and ANPs (**b**). Inserts: histograms of PSDs; lines give fitting of PSD with lognormal distribution function.

Both MNPs and ANPs embedded into gel-based substrates were synthesized by the LTE method, which provided their basic similarity despite different chemical compositions. Figure 1 shows TEM images of MNPs and ANPs with histograms of particle size distributions (PSD) given in the insets, where Pn (d) is the number average lognormal distribution function.

In both cases, the shape of nanoparticles was very close to being spherical, which was the result of their condensation from the vapor phase. The histograms were plotted as a result of the graphical analysis of 2572 images in the case of MNPs and 2206 images in the case of ANPs. In both cases, the diameter of nanoparticles fell within the 5–50 nm interval with a probability maximum of 14 nm. The histograms were nicely fitted by a lognormal distribution function:

(1) $$P_{n}(d)=\mathrm{Cexp}\left(-\frac{1}{2}{\left[\frac{{\ln(d/d}_{0})}{\sigma }\right]}^{2}\right)$$

Parameters of the distributions are given in Table 1; they were very close to each other. Using the PSD parameters the values of number-average and weight-average diameter could be calculated as the first and the third moments of the distribution. These values are given in Table 1. Summarizing this set of data, we may conclude that from the viewpoint of their shape and characteristic dimensions, MNPs and ANPs were very similar. The similarity preserves concern their properties in water suspensions.

The crystal structure of MNPs corresponded to single-phase maghemite with an inverse spinel cubic lattice (period a = 0.8357, 3 nm), space group Fd3m). The average size of the coherent diffraction domains was 12 nm estimated using the Scherrer approach [17]. The crystal structure of ANPs was γ-Al_2_O_3_ with a cubic lattice (period a = 0.7924, 7 nm). The average size of the coherent diffraction domains in ANPs was 17 nm.

The specific surface area (S_sp_) of nanoparticles, determined using low-temperature adsorption of nitrogen (BET method), was 93 m^2^/g for MNPs and 78 m^2^/g for ANPs. These values could be used for the evaluation of the average diameter of particles according to the following Equation (2) [18], which relates the diameter of a sphere to its surface (S) and density (ρ):

(2) $$d_{\mathrm{BET}}=\frac{6}{{\rho S}_{\mathrm{sp}}}$$

The crystallographic density of particles was 4.6 g/cm^3^ in the case of MNPs, and it was 3.6 g/cm^3^ in the case of ANPs. Given these values and the values of the specific surface area, the average diameter of MNPs was evaluated to be 14.0 nm, and the diameter of ANPs was 21.4 nm.

The specific feature of metal oxide nanoparticles synthesized by the method of gas-phase physical dispersion is their self-stabilization in water suspensions [19]. It means that stable suspensions of these nanoparticles in water can be prepared without using special stabilizers. The basic reason for that is the formation of traces of nitrogen oxides during the evaporation of the precursor oxide target in the air. This process is initiated by high temperature (approximately 10^4^ K) at the laser spot on the surface of the target during its evaporation. Nitrogen oxides then provide traces of metal nitrides on the surface of condensed nanoparticles. In water suspensions, nitrides dissociate, and the surface gets a net positive electrical charge due to the metal ions while nitride ions migrate to the water medium as counterions of the double electrical layer. It provides the colloidal stability of the suspension, which can be characterized by the value of the zeta-potential of the suspension. The values of zeta-potential for the self-stabilized suspensions of MNPs and ANPs are given in Table 1. They are positive both for MNPs and ANPs and close to each other. It indicates that the physical properties at the surface of these two batches of nanoparticles are likely common.

Meanwhile, self-stabilization of suspension of LTE nanoparticles is a limiting factor, and it can not provide the colloidal stability of these suspensions in the solutions with high ionic strength, which is typical in biological systems. Therefore, suspensions for biomedical applications were additionally stabilized by sodium citrate. Citrate ions adsorb on the surface and reverse their electrical charge from positive to negative; sodium cations become the counterions of the double electrical layer. The colloidal stability of the citrate-stabilized suspensions increases. Table 1 gives the values of zeta-potential in citrate-stabilized suspensions of MNPs and ANPs, which were used for the preparation of composite gel-based substrates for cell culture. In both cases, zeta-potential was highly negative, indicating that in both cases, the suspensions were very stable, and their colloidal properties were much alike.

**Figure 2:**
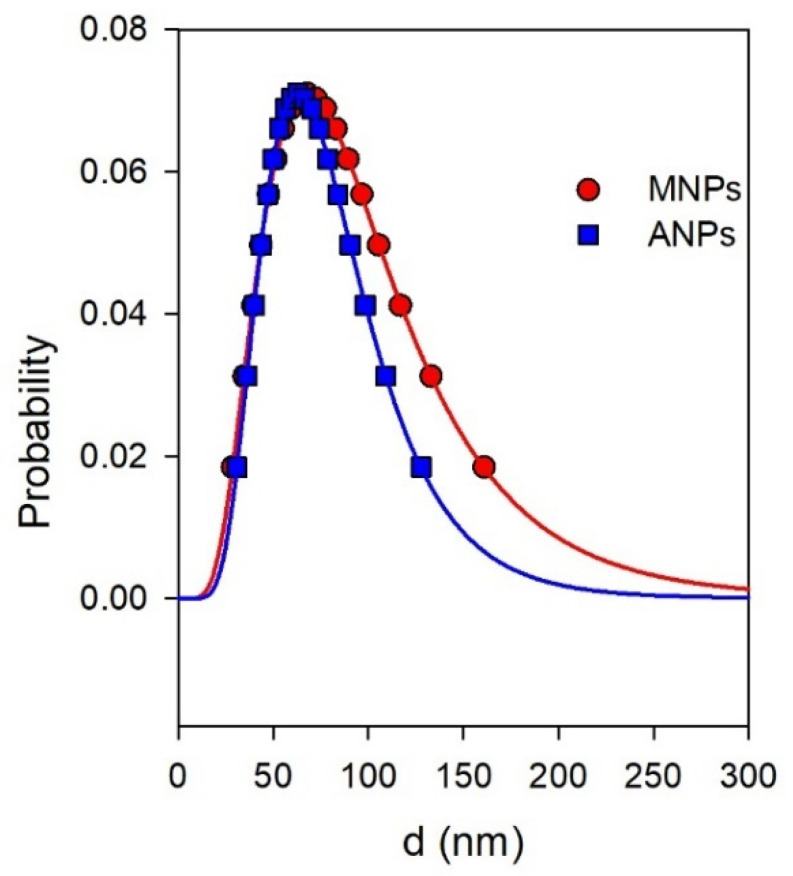
PSD of MNPs and ANPs in water suspensions measured by DLS. The probability function corresponds to the intensity of dynamic light scattering related to the fraction of nanoparticles with a certain diameter.

Figure 2 shows the PSD plots obtained by DLS for the suspensions of MNPs and ANPs in water. It is noticeable that the plots were very close to each other. The median of the PSD, which is commonly referred to as the hydrodynamic diameter (d_hd_) of a nanoparticle in a suspension, is given in Table 1 for the suspensions of MNPs and ANPs. The hydrodynamic diameter is an apparent value, which is the product of the Einstein equation for the diffusion coefficient. The diffusion coefficient is a value, which is measured by DLS. It is noticeable that the hydrodynamic diameters of MNPs and ANPs in their suspensions were close to each other. 

The average hydrodynamic diameter of the ensemble of nanoparticles is related to the fifth moment of PSD, which is given in Table 1 as the intensity-average diameter d_i_. There was a certain difference between the values of d_hd_ and d_i_. This difference partly stems from the solvation layers on the surface of nanoparticles in water medium. In addition, some small aggregates of nanoparticles still could remain in the suspension after centrifuging. The values of the fifth moment of distribution are highly sensitive even to the presence of a small number of aggregates. However, their total fraction is not dominant.

Thus, the analysis of the properties of MNPs and ANPs shows that these two batches of LTE nanoparticles are very similar concerning their shape, dimensions, surface properties in water suspension, and colloidal stability. The only difference between them is in their magnetic properties.

### 3.2. Magnetic Properties of Fe_2_O_3_ and Al_2_O_3_ Nanoparticles

**Figure 3:**
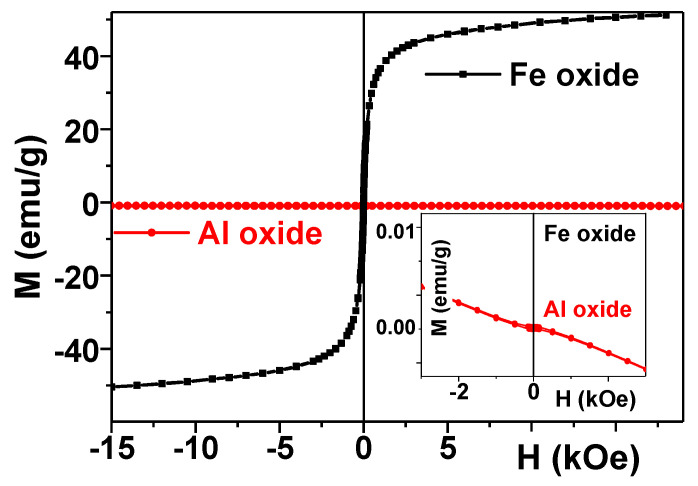
Magnetic hysteresis loops of Fe_2_O_3_ and Al_2_O_3_ LTE as-prepared nanoparticles. Inset shows the same responses in low magnetization scale.

Figure 3 shows magnetic hysteresis loops M(H) of dry as-prepared MNPs and ANPs. One can see a huge difference in their magnetic responses. γ-Fe_2_O_3_ magnetic nanoparticles are a well-known and well-studied biocompatible magnetic nanomaterial with high saturation magnetization. As expected for superparamagnetic nanoparticles of the size under consideration, the saturation magnetization (M_s_) was significantly reduced in comparison with the bulk case [20]. The S-shape of the hysteresis loop and very low coercivity at room temperature are typical features of superparamagnetic MNPs. In fact, the saturation was not achieved in a magnetic field of 19 kOe, but a rough estimation of the magnetization value in the field of about 19 kOe confirmed that it was consistent with the values for M_s_ previously obtained for MNPs of this size.

ANPs had a clear diamagnetic response (linear magnetization dependence on the value of the applied magnetic field with a negative slope and very small value of the resulting magnetic moment), as expected for this kind of material. Again, the difference was huge for the scale appropriate for M(H) hysteresis loop of γ-Fe_2_O_3_ magnetic nanoparticles, and the magnetic field dependence of the magnetization of Al_2_O_3_ nanoparticles was not visible. However, it became noticeable at a low magnetization scale (Inset Figure 3).

### 3.3. Structure and Magnetic Properties of Composite Gels

We have focused on the properties of the suspensions of nanoparticles because these features strongly determine the arrangement of the embedded NPs in the composite gels. Currently, there is no reliable experimental method for the direct observation of the arrangement of the embedded nanoparticles in swollen composite gels. Any microscopic technique presumes certain dehydration of the gel, which inevitably would disturb its intact structure. In this respect, we have to rely somehow on the indirect features of the synthetic procedure and the macroscopic properties of the fabricated composite gels.

As shown above, the precursor suspensions of MNPs and ANPs were almost de-aggregated and contained mostly individual nanoparticles with a fraction of small aggregates. These suspensions were transparent, which meant that there were no moieties larger than the wavelength of the visual light—approximately 600 nm. Moreover, there was no visual opalescence in the suspensions, and consequently, the limit might be lowered down to a quarter of a wavelength, which was approximately 150 nm. This estimation of the upper limit in the characteristic dimension of moieties present in the suspensions correlated well with the PSD given in Figure 2. 

**Figure 4:**
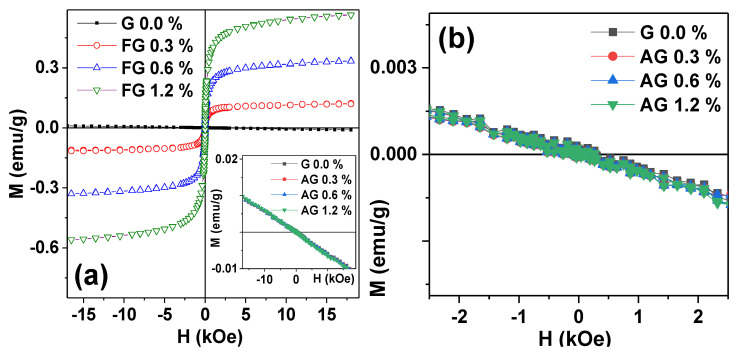
Magnetic hysteresis loops of gels and gel-based composites: MNP-based (**a**) and ANP-based (**b**) materials. G: blank gel; FG: Fe_2_O_3_-based ferrogels; AG: Al_2_O_3_-based gel composites.

During the synthesis, the visual appearance of the precursor suspensions did not change. They remain transparent at the stage of the mixing with reactants and at the stage of gelation as well. Visually, the gels look the same as the precursor suspensions. It made us assume that the distribution of the nanoparticles that were established in the precursor suspensions preserved, in general, in the resulted composite gels. Figure 4a shows the general view of all gel-based substrates. 

The mesh size of the PAAm network in composite gels was estimated based on the value of the swelling ratio of gel. The swelling ratio (α) is given by Equation (3):

(3) $$\alpha =\frac{m_{g}{-m}_{\mathrm{dr}}}{m_{\mathrm{dr}}}$$

where is m_g_ the mass of a gel sample and m_dr_ is the mass of the dry residue after the gel sample is dried to the constant weight.

However, in composite gels the value of m_dr_ includes both the mass of the dry polymeric network and the mass of the embedded solid nanoparticles. To obtain the swelling ratio of the polymeric network (α_p_), the correction should be done according to Equation (4):

(4) $$\alpha_{p}=\frac{\alpha }{1-\omega }$$

where ω is the weight fraction of solid nanoparticles in the dry residue.

The swelling ratio of composite PAAm gels determined using Equations (3) and (4) was found to be 12.6 ± 0.4 both for gel series with MNPs and ANPs independently of the content of nanoparticles. 

The swelling ratio of a hydrogel network is, in other words, the water uptake of the network, i.e., the amount of water that the network can hold within it. Its value, in principle, depends on the mesh size of the network and the energy of interaction between water and polymeric sub-chains. This dependence is given by the Flory–Rehner equation [21,22]. Using the equation, one can calculate the average number of monomer units in the sub-chain of gel network based on the value of the swelling ratio according to Equation (5):

(5) $$N_{c}=\frac{V_{1}({0.5\alpha }_{0}\alpha_{p}^{-1}{-\alpha }_{0}^{1/3}\alpha_{p}^{-1/3})}{V_{2}{(\ln(1-\alpha }_{p}^{-1}{)+\alpha }_{p}^{-1}{+\chi \alpha }_{p}^{-2})}$$

where N_C_ is the number of monomer units among cross-links of the network; V_1_ and V_2_ are the molar volumes of water and of PAAm, respectively; χ is Flory–Huggins parameter for a binary interaction between water and PAAm; α_0_ is the swelling degree of PAAm gel as provided by the composition of the reaction mixture in the synthesis. We used V_1_ = 18 cm^3^/mol (water), V_2_ = 56.2 cm^3^/mol (PAAm) and χ = 0.12. The last two values were calculated by means of quantum mechanics molecular modeling software package CAChe7.5. As it was given above, the equilibrium swelling ratio was 12.6; the swelling ratio of as-synthesized gel was 10. Given these values, the average number of monomer units among cross-links of the network according to Equation (5) was found to be N_C_ = 64.

Considering that PAAm sub-chains of the network are random Gaussian coils with hindered rotation, one can evaluate the mean square distance between adjacent cross-links of the network according to Equation (6) [23]:

(6) $$\langle R^{2}\rangle {=\mathrm{Na}}^{2}\frac{\left(1-\cos\;J\right)}{\left(1+\cos\;J\right)}\frac{\left(1-\cos\;f\right)}{\left(1+\cos\;f\right)}$$

where N is the number of bonds in the polymeric sub-chain, *a* is the bond length, *θ* is the bond angle, and *φ* is the angle of hindered rotation. In a PAAm polymeric chain, the bond length *a* = 0.154 nm for the ordinary C–C bond, the bond angle *θ* = 109.5°, the angle of hindered rotation *φ* = 120°, and N = 2N_C_. It is two-fold larger than N_C_ as it includes the bonds in monomer units and bonds between them. Given these values, the average distance among the cross-links according to Equation (6) was found to be 4.3 nm. This value was the effective mesh size of the PAAm network in the composite gels. It was evident that the mesh size of the network is much smaller than the characteristic dimensions of MNPs and ANPs (see Table 1). Thus, we may conclude that in both series, nanoparticles were entrapped in the gel network and were not able to leave it or to migrate along it. 

The substantial difference between these two series of gel-based composites was in their magnetic properties. The main methodological advantage of the present study is the employment of the same synthesis technique for the fabrication of two different types of nanoparticles, which we denote as ferromagnetic (iron oxide) and non-ferromagnetic (aluminum oxide). Here we keep in mind the most general division of magnetics is in ferro-, antiferro-, para-, and dia-magnetics [20]. The bulk magnetite is a ferromagnetic material with rather high saturation magnetization [20]. However, in the case of nanosized particles, the saturation magnetization of iron oxide MNPs becomes strongly dependent on their size [11]. Alumina is a dia-magnetic material. The properties of as-prepared MNPs and ANPs were discussed above. Now let us analyze the magnetic properties of gel-based composites measured at room temperature (see Figure 4). 

Figure 4 gives the comparison of magnetic properties of gel-based composites with MNPs and ANPs. An appropriate scale helps to visualize the difference in the best way. One can see that, as expected for the mainly water containing sample, hydrogel (G 0.0%) had quite a weak diamagnetic signal, which was much lower than the signals of MNPs in the concentrations under consideration.

Despite small concentrations of MNPs the magnetic properties of ferrogels were very different for the range of selected concentrations (Figure 4a); the higher is the concentration, the higher is the magnetic moment. The shape of the hysteresis loops of ferrogels was quite similar to the shape of the MNPs, typical for superparamagnetic particle responses with close to zero coercivity. S-shaped geometry but a lower saturation magnetization value was observed in comparison with MNPs.

Meanwhile, for the same concentrations of the ANPs in gel-based composites (Figure 4b), we could not see the same tendency; at the available accuracy of magnetic measurements, the magnetic responses of all composite gels with embedded ANPs showed very similar typical diamagnetic linear responses, which means that the magnetization declined with respect to the applied external magnetic field with close values of the negative slope. 

### 3.4. Effect of NPs Concentration on the Young’s Modulus and Electrical Potential in Composite Gels

**Figure 5:**
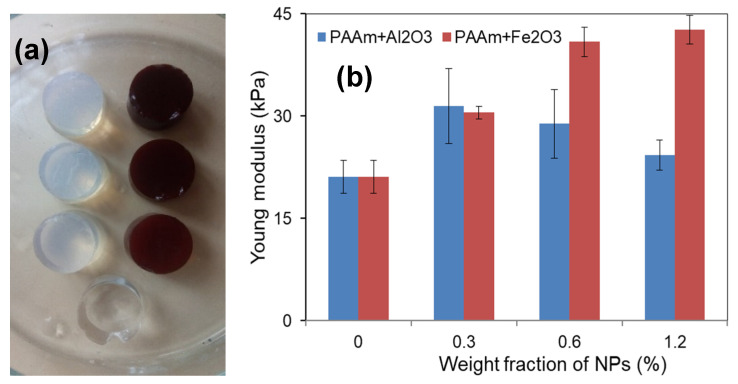
General view of gel-based samples: (**a**) ANPs-based composites, (**b**) MNPs-based composites, and the completely transparent sample is a blank gel; the diameter of the gel samples was equal to 13 mm. The values of Young’s modulus for PAAm hydrogels filed with different concentrations of MNPs or ANPs. Data presented as mean value and standard deviation bar (*n* = 5).

Figure 5 shows the general view of the gel-based composites with different content of the embedded NPs (Figure 5a) and the effect of iron oxide or aluminum oxide NPs concentration on the value of Young’s modulus in the PAAm gels (Figure 5b). One can see that the addition of particles at a minimum concentration (0.3%) to the polymer network resulted in a significant increase in the composite rigidity. Moreover, the contribution of MNPs or ANPs to the modulus at the lowest concentration in the gel network was approximately the same. 

At higher content of nanoparticles in composite gels, their concentration connection with Young’s modulus was different. While in the case of MNPs the rigidity of composite gels increased further with the concentration increase, the gels filled with ANPs showed a tendency to decrease Young’s modulus. The increase om the ANPs concentration from 0.3% to 1.2% was accompanied by the subsequent decay of rigidity of composite gels so that at ANPs concentration of 1.2%, the modulus was significantly smaller than that one at 0.3%. At the same range of MNPs concentrations, the rigidity of ferrogels was gradually increasing. Noteworthy, at nanoparticle concentrations of 0.6% or 1.2%, the Young’s modulus of composite gels with embedded MNPs was significantly larger in comparison with the composite gels with embedded ANPs. 

**Figure 6:**
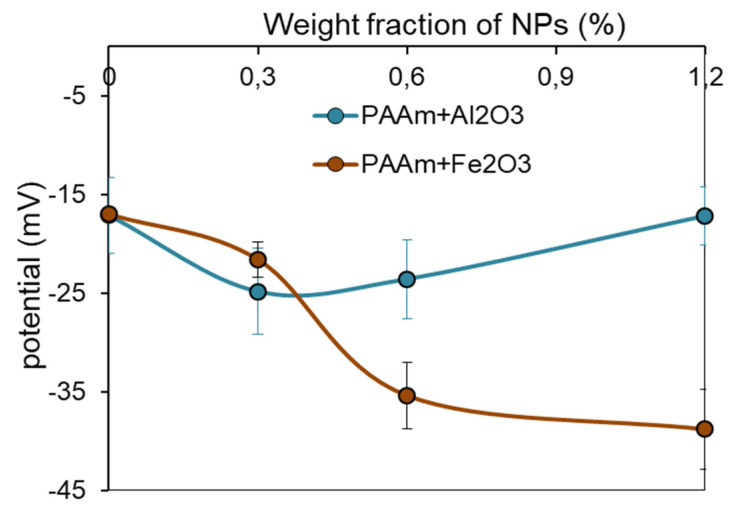
Dependence of electrical potential in gels on the concentration of Fe_2_O_3_ or Al_2_O_3_ nanoparticles. Data presented as mean value and SD bar (*n* = 7).

Figure 6 shows the general view of the gel and composite gels and the dependence of the electrical potential in gel-based composites on the concentration of MNPs or ANPs. One can see that the addition of nanoparticles to the PAAm gel at a minimum concentration (0.3%) resulted in an increase in the potential in absolute value so that both composite gels became more electronegative to approximately the same extent. A further increase in nanoparticle concentration led to a different change of the electrical potential for the gels with MNPs or ANPs. In gels filled with MNPs, the potential became progressively negative with the increase in MNPs content. At the same time, the opposite trend appeared in gels filled with ANPs. While the content of ANPs further increased, the negative values of the potential diminished. Furthermore, the differences in potential for the two series of composite gels were greater for the higher concentration of the particles.

### 3.5. Effect of NPs Concentration on the Proliferation Activity of Cells

**Figure 7:**
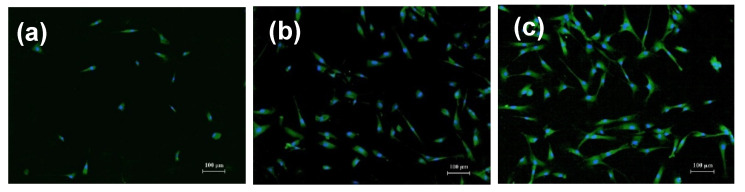
Fibroblasts on the surface of gel-based substrates. Cells were cultured for 96 h and fixed for fluorescent microscopy. Cell nuclei were stained with DAPI (4,6-diamidino-2-phenylindole), the cytoplasm was stained with pyrazolone yellow. (**a**) blank gel substrate; (**b**) gel-based substrate with 1.2% Al_2_O_3_ nanoparticles; (**c**) gel-based substrate with 1.2% Fe_2_O_3_ nanoparticles.

Figure 7 shows typical examples of the human dermal fibroblast cultures grown onto the surface of different PAAm gel-based substrates after appropriate staining. The first important observation is that the cell culture under consideration could be successfully grown onto all kinds of composite substrates described above. The second conclusion was that MNPs and AMPs made essential positive contributions to cellular proliferation in the described conditions.

Table 2 displays the results of fibroblasts counting onto the surface of the tested gel-based substrates after four days of incubation. The data were obtained based on the analysis of nine fields of view for each biological sample, and they are presented as X and m, where m is the error of the mean (X, *n* = 54). To assess the viability evaluation of the used fibroblasts, cells were also seeded onto the standard substrate—tissue-culturing polystyrene (TCPS). At the end of incubation, the number of cells was 21,000 ± 1000 per cm^2^, which corresponded to good cell viability.

According to the obtained data, the addition of both MNPs and ANPs to the PAAm gel network led to a significant and almost the same increase in the proliferation activity of fibroblasts. Importantly, at the initial NPs concentration of 0.3%, both of the gel-based composites with MNPs and ANPs had similar Young’s modules and electrical potentials (see Figure 4 and Figure 5). In general, the effect of NPs concentration on the cell proliferation rate grew gradually in both series of composite gels.

The contribution of specific NPs to cell proliferation rate must be compared in the gel-based substrates with approximately the same mechanical and electrical properties. According to the results presented above (see Figure 4 and Figure 5), the gels filled with ANPs of 0.3% and 0.6% had a similar Young’s modulus and electrical potential. In the case of ferrogels, these are substrates with MNP concentrations of 0.6% and 1.2%. One can see in Table 2 that the gain of the increase in cell proliferation rate from 0.3% to 0.6% for gels with Al_2_O_3_ NPs, and from 0.6% to 1.2% for gels with Fe_2_O_3_t NPs, was significant and approximately the same. 

## 4. Discussion

In the present study, the biological activity of cells was investigated for two types of gel-based composites: PAAm hydrogels filled with γ-Fe_2_O_3_ or Al_2_O_3_ nanoparticles in different concentrations. Despite the difference in the chemical composition of the nanoparticles (alumina or maghemite), these two series of substrates were similar in many senses. Both were based on PAAm gel with the same concentration of monomer (1.6 M) and the same concentration of cross-linker (1:100 molar ratio to monomer). The nanoparticles were synthesized by the same method (LTE) using the same laboratory installation; both types of nanoparticles were spherical, non-agglomerated, and had close characteristic dimensions (10–40 nm) and parameters of PSD. In both series of gel-based substrates, the inner distribution of NPs in the PAAm matrix was approximately the same. 

**Figure 8:**
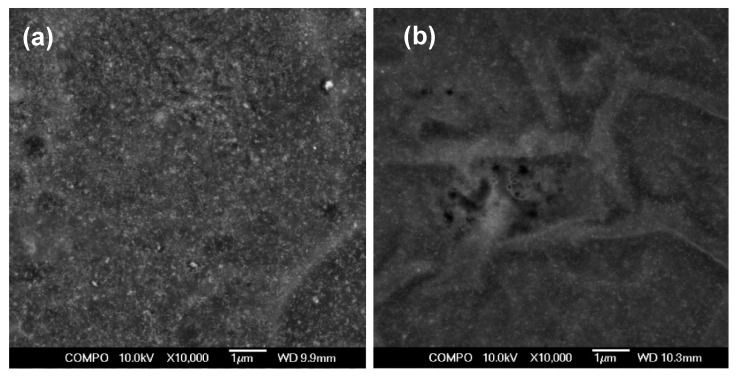
General view of the surface of dry of PAAm gel-base composites with Fe_2_O_3_ (**a**) or Al_2_O_3_ (**b**) nanoparticles (1.2 wt.%). Scanning electron microscopy.

Figure 8 shows typical SEM images of the dried surfaces of MNPs and ANPs based composites. We provide low magnification data in order to emphasize the difficulties of the structural investigation of these types of composites. One can clearly see that the dehydration process resulted in the appearance of strong stresses and surface relief. Even so, these observations confirm the expectation that at a large scale, both MNPs and ANPs were distributed in quite a homogeneous manner without the formation of very large agglomerates.

As discussed in previous work [10,13], as the potential applications of gels and composite gels are under active development, at present, there is a special need for the development of a new technique for gel-based composites characterization. Existing techniques are not adequate for quantitative evaluation of the structural arrangement of the MNPs or ANPs inside the gel. However, understanding the internal structure of filed gels is important, and we used earlier developed techniques for the evaluation of the structure of dried composites with MNPs and AMPs [10,13]. 

The magnetic characterization revealed that two series of gel-based composite substrates were very different in terms of their magnetic properties. While gels with MNPs showed ferromagnetic behavior with magnetization proportional to the content of iron oxide, gels with ANPs are diamagnetic, it is clear that any difference in the cell cultivation results of gel substrates with embedded ANPs could not be assigned to the difference of magnetic properties of gel composites with different concentration of ANPs.

The possible influence of magnetic interactions between iron oxide nanoparticles could be taken into account for the gel-based composites with embedded MNPs. However, as in the present study, all cell proliferation experiments were performed without the application of the external magnetic field. Although it is unlikely that they might provide a substantial contribution, this point requires further investigation as many other cases involving small magnetic fields [24]. From the low field behavior of magnetization of MNPs based composites, one can estimate the contribution of the stray fields created by the iron oxide MNPs in the field of a few Oe as almost negligible. In the future, it would be interesting to make an evaluation of the cell proliferation rate in the same conditions as in the present case but under application of the external magnetic field of the order of 100 Oe (at least).

The electrical potential and the mechanical properties of composite gels were almost the same at the lowest content of NPs (0.3%), no matter whether NPs were magnetic (maghemite) or diamagnetic (alumina). Meanwhile, different trends in Young’s modulus and in the electrical potential were found as the content of NPs increased (see Figure 5 and Figure 6). In the case of gel-based substrates with maghemite, the modulus and the electrical potential enlarged if the content of NPs increased from 0.3% to 1.2%. The opposite trend of the diminishing of the modulus and the electrical potential was found in the case of gel-based substrates with alumina. The decrease in Young’s modulus with the concentration of ANPs in gel-based composites cannot be easily explained. Most likely, it might be the result of some specific interaction of ANPs with the PAAm network, or it might stem from the different trends of aggregation of MNPs and ANPs inside the gel structure. A qualitatively similar effect was reported in [25] for the influence of alumina nanoparticles (mean diameter of 30 nm) on the elasticity of composite PAMPS/PAAm gels. For now, we cannot give a plausible explanation for the underlying mechanism but can only state the difference in the mechanical behavior between two series of gel-based composites. 

However, despite the marked difference in the magnetic properties, the electrical potential, and the elasticity between magnetic and diamagnetic gel-based substrates, the proliferation of human fibroblasts on these two platforms revealed qualitatively the same result. In both series of gel-based substrates, the increase in NPs content resulted in the enhancement of the proliferation activity of fibroblasts. In meant that the magnetic properties of maghemite NPs did not play a significant role in the determination of the biological activity of fibroblasts on the PAAm gel-based substrates with embedded NPs. In other words, a key factor other than the magnetic properties of the gel-based substrates governs the biocompatibility of nanostructured composites.

Fibroblasts are a type of mechanically sensitive cells, which are able to accept and transform mechanical signals for their vital activity [26,27,28]. The feasible mechanism of the transduction of mechanical signals into the cell involves heterodimeric trans-membrane receptors, which are referred to as integrins [26,29]. Clusterization of the integrins at the cell membrane in response to the signal triggers the trans-membrane accumulation of the 20 mediators of the signal transduction, including cytoskeleton proteins, which regulate the functioning of the cell by means of the activation of corresponding genes [26,30,31]. It is also assumed that the mechanical signals are transmitted into the cell through the mechanically gated cationic channels through the stretch-activated channels (SAC channels) [32].

In vitro experiments have shown that the surface geometry pattern played a significant role in the realization of the mechanical transduction phenomenon in cells together with chemical composition, wettability, surface charge, elasticity, and other factors [33,34,35]. Thus, it was shown that the specific geometry pattern of the surface of solid nanocomposite materials (dimples, bumps, their shapes), including the characteristic dimensions of the roughness and its periodicity, could initiate the proliferation and the differentiation of cells [36,37,38]. Furthermore, it was demonstrated that the specific patterns of the surface of a nanocomposite initiated the corresponding specific signal routes triggering the activity of certain genes [30]. Although the observation with dried composites was quite preliminary and requires further investigation, the difference in the physical properties (for example, magnetostriction) of MNPs and ANPs may be the reason for the fine surface structure formation.

The visualization of the intact surface geometry pattern of gel-based composites is still the unsolved challenge for the conventional microscopic approaches due to the presence of a large amount of solvent in gel interior structure. Such methods of preparation as freeze-drying or vacuum drying strongly disturb the surface of samples. Therefore, the results of microscopic studies like AFM or SEM do not characterize all details of the surface geometry explicitly. Meanwhile, the results of SEM and TEM confirm indirectly the heterogeneity of the surface of nanocomposite gels [39,40]. For instance, such data were reported in our earlier published work [13], and here, we applied this technique for the composites with diamagnetic nanoparticles. One of the consequences of the change of the type of nanoparticles was much lower contrast in SEM studies; as γ-Fe_2_O_3_ were characterized by higher “electronic density”, i.e., they interacted more actively with the electronic beam, they look much brighter (Figure 8a), and provide much higher contrast in comparison with Al_2_O_3_ nanoparticles inside the dried composite (Figure 8b). 

Hypothetically, the effect of nanoparticles on the surface geometry can stem both from the absence and from the presence of NPs at the gel interface. For instance, NPs located in the outer layers of the gel could disentangle from the networks and move to the liquid phase, leaving voids in the surface layer. In the case of gel-based composite substrates in the present study, these voids are likely the size of the NPs, which was 10–40 nm, and were separated by approximately 150 nm one from another (evaluation was done for the weight fraction of nanoparticles equal to 1%). Thus, the surface of the gel substrate might be covered with small dimples separated by ca. 0.15 μm.

It is also feasible that NPs, which disentangle from the gel network, do not move to the bulk of the liquid phase but provide the adsorption layer at the surface. There are certain grounds for such supposition. It was shown in previous work [41] that the interaction of polyacrylamide macromolecules with the surface of iron oxide nanoparticles is energetically favorable. Alumina nanoparticles also have high adsorption potential and high catalytic activity at the surface due to a large amount of Al-OH moieties at the surface [42,43]. Hence, it would be reasonable to assume that there are adsorption forces at the gel interface, which can immobilize nanoparticles at the surface. Due to the adsorption of nanoparticles, the roughness of the surface enhances. The exudation of nanoparticles from the gel network and their adsorption on the surface might not be the alternatives, but both could contribute to the surface micro-roughness of gel-based composites. In addition, cell cultivation takes place in the solutions of high ionic strength; the presence of the immobilized at the surface nanoparticles can change the diffusion conditions affecting in this way the cell culture grows.

The mechanism of the bonding of the mechanically sensitive receptors of cells to the heterogeneous surface patterns of nanocomposites is not elucidated yet. Supposedly it may be provided by a certain packing of integrins, which match the characteristic dimensions of nanostructures, like the diameter of nanotubes [44,45]. A model was introduced which described the adhesion of cells to the nanostructured surface based on the energy of deformation of the receptors due to their interaction with the surface geometry pattern [35]. Electrostatic interactions between receptor molecules and nanostructures at the surface might also be feasible [46,47].

Besides, we may suppose that the adhesion of cells to the scaffold can be mediated by proteins, which are the components of the medium for the cell culturing and better adsorb at the rough surface. It was reported that even a small amount of vitronectin and fibronectin at the surface of ferrogel substantially promoted the adhesion of cells due to the presence of a specific amino acid sequence (Arg-Gly-Asp) in their chemical structure, which is favorable for the interaction with membrane receptors of fibroblasts [48,49]. Similar results were obtained for the adhesion and proliferation of human fibroblasts on the surface of Al/Al_2_O_3_ bi-phasic nanowires (NWs) [50]. 

Thus, it is reasonable to interpret the results obtained in the present study from the viewpoint of the structuring of the surface of the gel-based composites by the exudation or/and adsorption of nanoparticles. The mechanisms of the surface structuring are likely the same for the composite PAAm gels filled with alumina or magnetite, and therefore the effect of gel-based composites on the adhesion and proliferation of human fibroblasts might be the same as well, i.e., gel-based composites provide a heterogeneous surface geometry pattern, which is favorable for the adhesion of cells.

In general, the obtained results showed that in the elaborated experimental conditions, the proliferation activity of human fibroblasts on the surface of gel-based composites did not depend on the magnetic properties of the embedded nanoparticles. It is worth mentioning that this finding was not influenced by the fact that the gel-based composites of the magnetic and diamagnetic origin significantly differed in their Young’s modulus and electrical potential. It may be taken as additional support for the hypothesis that the surface geometry pattern has a key role in the biocompatibility of gel-based composites.

In the meantime, there is quite a lot of studies that address ferrogels as prospective magnetically controlled composites for applications in tissue engineering, regenerative medicine, field-assisted drug delivery, and magnetic biosensing. Various polymers: synthetic, biological, and their blends are used as matrices for these composites. Iron oxide magnetic particles embedded in composite ferrogels also vary in dimensions, chemical compositions, synthetic routes, and other parameters. In general, results obtained in these studies confirm good biocompatibility of ferrogels, and the achieved level of magnetic properties of these materials makes them especially attractive for magnetic field-assisted drug delivery and magnetic biosensing [5,51]. The present combination of the composites based on superparamagnetic and diamagnetic nanoparticles can be of special interest in the understanding of a high-frequency response of polymer matrix with variations of dielectric constants contributions in the formation of magnetoimpedance responses of the gel-based composites [51].

The present study is a special step allowing a comparative evaluation of the contributions of MNPs and ANPs. This comparison finally became possible because of our research works related to the biological activity of cells at hydrogels and ferrogels in similar experimental conditions [8,10,11,12,13]. In this series of experimental studies, we used human fibroblasts taken from one patient, magnetic nanoparticles of iron oxide Fe_2_O_3_ from the same batch, PAAm gel with the same networking, the same procedures, and experimental techniques. According to these studies, the increase in MNPs content in ferrogel always led to the reliable enlargement of the density of cells monolayer at the surface of magnetic composites. Similar results were obtained in the present research, which compared the biocompatibility of gel composites with embedded magnetic iron oxide nanoparticles and embedded non-magnetic alumina nanoparticles, which were very close in terms of their shape and dimensions.

## 5. Conclusions

Two series of non-agglomerated spherical nanoparticles of 20–40 nm in diameter were fabricated by laser target evaporation technique: superparamagnetic Fe_2_O_3_ or diamagnetic Al_2_O_3_ nanoparticles. Composite polyacrylamide gels with Fe_2_O_3_ or Al_2_O_3_ embedded nanoparticles were synthesized, aiming to study the magnetic contribution to the ferrogel biocompatibility. The proliferative activity of human dermal fibroblast cell cultures on the surface of these gel-based composites was estimated. The concentration of the fillers in the gel was fixed at 0.0, 0.3, 0.6, or 1.2 wt.%. Mechanical, electrical, and magnetic properties of the composites were characterized by the dependence of Young’s modulus, electrical potential, magnetization measurements on the content of embedded nanoparticles. The fibroblast monolayer density on the surface of composite substrates after 96 h of incubation without application of external magnetic field was evaluated for estimation of the composites biocompatibility. It was found that regardless of the nature of the nanoparticles, the increase in their concentration in the composite provided a parallel increase in the cell proliferation on the surface of composite substrates.

The main conclusion of the present study is the statement that the biological activity of cells on the surface of composite gels does not depend on the magnetic properties of nanoparticles, at least in the elaborated experimental conditions. In other words, the obtained results exclude a significant contribution of the magnetic field provided by magnetic nanoparticles inside ferrogel and near its surface on the biocompatibility of magnetic gel composites in the conditions under consideration.

In general, the present study has more methodological than applied value. It is addressed toward the search of a key determinant of FG biocompatibility. In a series of preliminary experiments performed under the same experimental conditions, we tried to exclude systematically the indirect contribution of various factors that can determine cell biological activity at the surface of ferrogels, in particular, the mechanical properties and electrical potential of composites. In this study, we excluded the direct contribution of the FG magnetic effects in very low magnetic fields of the order of terrestrial magnetic field values. Finally, we assumed that the FG biocompatibility is most likely associated with the effect of the particles on the composite surface. At the same time, the results obtained can be useful in the design of gel-based composites for cell technologies. In this context, the application of ferrogels with the use of an external magnetic field is of the greatest interest. From our point of view, research in this direction will make it possible to optimize the principles of controlling biological processes in cell cultures grown onto FG. Special attention should be paid to one very important aspect of this study: iron oxide magnetic nanoparticles from the same batch were used in previous experiments, ensuring a very good basis for comparison of the results obtained for Fe_2_O_3_ and Al_2_O_3_ systems fabricated by the same technique and characterized in the same conditions.

## Figures and Tables

**Table 1:** Selected parameters of PSD for MNPs and ANPs in air-dry powder and water suspension.

Batch	Air-Dry Powder					Water Suspension		
	d_0_ (nm)	σ	d_n_ (nm)	d_w_ (nm)	d_i_ (nm)	d_hd_ (nm)	ζ_0_ (mV)	ζ (mV)
MNP	14.1 ± 0.4	0.46 ± 0.02	14.9	28.3	40.3	68 ± 3	35 ± 4	−66 ± 5
ANP	14.5 ± 0.4	0.35 ± 0.02	17.9	24.1	34.3	62.9 ± 0.4	40 ± 5	−59 ± 4

d_n_: number-average diameter, calculated at the first moment of PSD; d_w_: weight-average diameter, calculated at the third moment of PSD; d_i_: intensity-average diameter, calculated at the fifth moment of PSD; d_hd_: hydrodynamic diameter in suspension by DLS; ζ_0_: zeta-potential in self-stabilized suspension by ELS; ζ: zeta-potential in suspension stabilized with sodium citrate by ELS.

**Table 2:** Proliferation of fibroblasts on the surface of PAAm gels with different concentrations of Fe_2_O_3_ or Al_2_O_3_ nanoparticles.

Nanoparticles Concentration (wt.%)	Cell Monolayer Density (Cells per cm^2^)	
	PAAm 1:100 + Al_2_O_3_	PAAm 1:100 + Fe_2_O_3_
0.0	400 ± 100	
0.3	900 ± 100 *	1400 ± 400 *
0.6	1900 ± 300 *^#^	1400 ± 300 *
1.2	2400 ± 300 *^#^	2500 ± 400 *^†^

Symbols display significant differences with *p* < 0.05; *: between composite gels and blank gels (PAAm, concentration of NPs = 0.0%); ^#^: between Al_2_O_3_ composites (NPs = 0.6% or 1.2% and NPs = 0.3%, respectively); ^†^: between Fe_2_O_3_ composites (NPs = 1.2% and NPs = 0.3% or 0.6%, respectively).
